# Extracts and Abstracts

**Published:** 1889-03

**Authors:** 


### EXTRACTS AND ABSTRACTS.

***Transplantation of Testis from Perineum.*** —An interesting case is reported from the Hospital for Sick Children, London, and which came under the care of Mr. Edmund Owen. He says:

The condition of malposition of the testis in this case is of considerable rarity, known as ectopia perinæalis, one of the varieties of ectopia testis, the other being ectopia cruralis, in which the testis has left the abdominal cavity by the crural ring. It is usually the left testis which is displaced into the perineum, but the right may be in this abnormal position. Hutchinson met with a case in which both were so displaced, but this is the only one on record. The condition is diagnosed early in life, as it is usually congenital. Godard, however, recorded an instance of its descent at the age of twenty-five. Most of the other instances in which a late descent (or dislocation) has been supposed to have occurred are doubtful, as in the case of the soldier, aged twenty-four, on whom Partridge operated. It has, however, been unexpectedly met with by Ricord when epididymitis had set in, and by Zeis, who was about to perform lithotomy on a boy of fifteen. The cause of the malposition of the organ has been ascribed to irregularity in the attachment of the gubernaculum testis; in more than one of the cases it has been felt as the retaining-band; in others, this band has been seen at the operation for reposition and described as the gubernaculum. Symptoms referable to its presence in the perineum are usually the result of injury; but, as in Annandale's case, pain may be caused by walking or running, or, again, by sitting, and more especially by riding. Attempts to replace it in the scrotum by other than operative measures have usually proved abortive, and, as it is frequently well developed, it is not advisable to perform castration unless it be associated with congenital hernia, as in the case operated on by Surgeon Flanagan at Mhow, or on failure of the operation for reposition. The earlier operations for replacement were unsuccessful. Curling, in the case of a child, used wire sutures and a pad over the perineal sac; he found the testis difficult to transplant; suppuration followed, and the patient died at the end of a fortnight, from exhaustion and diarrhœa. Adams, who attended a child (aged eleven weeks) at home after operation, lost his case from erysipelas and phlebitis fourteen days later; inflammation extending along the funicular process. He secured the testis in the scrotum by means of a catgut stitch through the cut gubernaculum. Partridge, in the case of the soldier already referred to, used deeply placed subcutaneous sutures and applied a pad to the perineum. As a result of a consideration of the causes of failure in his case, Adams made certain suggestions, recommending that a child should be permitted to attain the age of two or three, and that the testis should be secured in the scrotum by means of sutures. The employment of the antiseptic treatment has been most successful. Marshall, in a boy of sixteen, tied the testis to the lowest part of the scrotum by means of a catgut stitch, and used antiseptic gauze; this case was successful. So was the operation when done by Annandale at an earlier period in a boy aged three years; a catgut stitch was passed through the testis and bottom of the scrotum, and antiseptic treatment employed. The success attained by Annandale and Marshall and in the following case will doubtless be imitated in the future, now that the principles of the operation are more fully recognized. Some years ago Mr. Owen brought forward the case of an infant with displaced right testis in which he did not recommend operative treatment. There are various questions of further interest suggested by the following case, such as the question of malignant disease in misplaced or undescended testicles, into which we have not space to enter. For the following reports we are indebted to Mr. R. C. Priestley, M.A., registrar.

Case 1. *Transplantation of testis.*—L. C——, aged two years and seven months, was admitted on September 28th, 1888. The child is small for his age, and is unable to talk, but his limbs and head are well formed. On examining the genital organs, the right testicle was found well developed, and in the usual situation; the left testicle was also of normal size, but was situated in the perineum, to the left of the median raphe; the cord of the left testicle was apparently as long as that of the right, as the two organs were on a level with each other. The left half of the scrotum was small, and drawn over to the right side. On Oct. 3rd, Mr. Owen made an incision over the left half of the scrotum, through which the misplaced testicle was reached, and freed from adhesions. There was no difficulty in placing the gland in the scrotum; but, as it obstinately slipped back into its original position, a deep suture was passed from one side of the left scrotum to the other, behind the testicle, which effectually maintained it in its new position. The edges of the wound were drawn together by a continuous suture, and drainage was provided for by a narrow piece of tissue inserted at the lower angle of the wound. The wound united readily, except at one spot, where a very small slough separated, and when the child quitted the hospital, a fortnight later, the left testicle appeared quite natural. The child has since been inspected, and the result of the operation has, apparently, left nothing to be desired.

It not infrequently happens that a testis which has failed to complete its descent, or has wandered into the groin or perineum, is imperfectly developed. In such cases it is a question whether it is deserving of surgical attention—that is, with a view to transplantation. If it be a fact, as some maintain, that the imperfectly developed gland is specially prone to malignant disease, it would, no doubt, be advisable to remove it without delay. And if, on the other hand, it is associated, as I have sometimes noticed, with a reducible inguinal hernia, ablation should also be practiced, so that the inguinal canal can be scarcely blockaded. It would be on merely sentimental grounds that, in such circumstances, any desire were felt for its preservation. But, given a healthy child with a well-developed and wandering testis, a simple operation such as that described above is not only justifiable. but, for every reason, expedient. As regards the nævus, I do not think that any other method of treatment could have given so prompt and thorough a result. Had it been treated by the old method of transfixation and ligature, the infant would not improbably have sunk exhausted on account of pain and suppuration. Moreover, the deepest parts of the mass could not have been destroyed by this means. Electrolysis could scarcely have dealt with it in less than three operations. In removing a nævus by the scalpel, it is necessary to keep well beyond the nodules of vascular fat which surround the spongy and growing mass. Then, with plenty of pairs of catch-forceps, the whole of the tumor can be cleanly removed without hæmorrhage or shock; and, as regards the operator, without anxiety or apprehension. —*The Lancet*, Feb. 9, 1889.

---

***How to Irrigate the Puerperal Uterus.***—In an article on "Irrigation of the Puerperal Uterus" (*Southern California Practitioner*, Feb., 1889) Dr. Walter Lindley says:

The instruments used are a fountain syringe holding a gallon, with a vaginal nozzle and a double uterine irrigation tube. The tube we use was made by Lentz, 18 North 11th street, Philadelphia. It is thirteen inches long, one and one-half inches in circumference, and is well curved. The openings for the entrance of fluid are small ($\frac{1}{8}$ inch diameter) and numerous and extend over about two inches of the upper surface of the end of the tube. Deep grooves on the sides and large openings ($\frac{3}{8}$x$\frac{7}{8}$ inch) into the return channel, extending over five inches of the under surface of the uterine end of the tube, provide for the free exit of the injected fluid. The larger the tube the less likely are we to have choking of the exit channel. A tube of smaller diameter is used to wash out the non-puerperal uterus after operations on its cavity, as curetting, etc.

First, instruments and hands are thoroughly scrubbed with soap and water, and soaked in tartaric-sublimate solution 1:500. The double tube must be boiled for half an hour before each irrigation, and afterward thoroughly cleaned with nail-brush. Clean out each hole carefully.

About three inches from the edge of the bed a small pillow is placed lengthwise, and over this an ample piece of rubber cloth so arranged as to form a spout passing into a bucket. The patient is placed across the bed, over the rubber cloth, the hips projecting slightly over the edge of the bed. The head and shoulders are comfortably supported by pillows; each leg is well wrapped in a separate blanket, the feet resting on two chairs placed widely apart. After the pudential hair has been removed by scissors and the external parts thoroughly cleaned, the vagina is thoroughly cleaned by injecting a gallon or more of warm water, through a hard rubber vaginal nozzle, the holes of which have been greatly enlarged by a penknife. After the vagina has been thoroughly cleaned, a quart of sublimate solution 1:4000 is allowed to run into it. Take care that the cleaning process is extended into all the folds and recesses of the vagina and cervix, by moving the nozzle in all directions. This must not be left to the nurse.

Now the forefinger of the left hand (which has, as a matter of course, been thoroughly disinfected, warmed and greased) is gently passed into the uterine cavity, the palm of the hand hugging the anterior vaginal wall, and if necessary the uterus being pressed gently downward. If any foreign matter, as clots or membranes are detected, the patient is etherized, the well-greased hand is inserted into the vagina, and one or two fingers into the uterus, which is gently but thoroughly emptied by repeated crooking motions of the fingers, adhesions to the endometrium being separated by gentle scraping with the finger nail.

The uterus having been thoroughly emptied, or having been found empty, the left forefinger is again inserted past the marked flexure always existing in the puerperal uterus at or near the internal os, and the double tube passed along it to or near the fundus. *No force must be used.* The procedure greatly resembles the passage of a sound into the male bladder. The flow is started before the tube is inserted to prevent the entrance of air.

Nothing but pure hot water should be used until the genital canal is thoroughly cleaned. Then a quart of tartaric sublimate solution 1:8000 may be used, and should be followed by more hot water.

The tube is allowed to remain for a few seconds, so that all fluid may drain from the uterus. The patient is then turned well over on her side, so that the vagina may empty itself.

The vaginal lesions should now be thoroughly dusted with iodoform (or if diphtheria exists the patches, according to Lusk, should be painted with equal parts of a mixture of persulphate of iron and compound tincture of iodine), and a hollow suppository holding half a drachm of powdered iodoform pushed well into the uterus.

*To Avoid Sublimate Poisoning:* 1. Where intra-uterine irrigation is used in the absence of sepsis, use no sublimate, but plain hot water or salt and water. 2. If the urine is albuminous and scanty, use no mercury. 3. If the urine is slightly albuminous and copious, or if the patient is profoundly anæmic, do not use more than a pint of a solution of 1:8000. 4. Always use tartaric acid and sublimate tablets or powders; dissolve thoroughly in a small quantity of water and mix carefully with a definite quantity of hot water in a pitcher, from which pour into the irrigator.[1] 5. Always use fountain syringe, and for the uterus a double tube, so as to insure the return of the solution. If for any reason the fluid fails to run out as fast as it flows in (if not through the reflux tube, by way of the channels at its sides), shut off the flow. The irrigator should not be raised more than three feet. 6. Precede by copious irrigation with hot water to wash out blood, etc., which may form with sublimate adhesive albuminous componds, which may in time be absorbed. Follow by a quart or two of hot water to insure the evacuation of all the sublimate solution. 7. For the uterus use a solution not stronger than 1:8000 and not more than a quart once daily. 8. For the vagina use a solution not stronger than 1:4000 and not more than a quart twice daily.

Irrigation used in the above way is, we believe, a practice almost devoid of danger. We have made more than one hundred and seventy-five irrigations with the double tube and fountain syringe, with no untoward results except in two cases an unimportant rise of temperature, and in one a severe but harmless chill, and even these slight accidents we feel certain might have been avoded by greater care. Yet irrigation of the puerperal uterus will always be a procedure requiring great care and judgment and some skill.

[1] Campbell, twenty-first and Pine streets, Philadelphia, makes the only really good tablet in the market. It is composed of sublimate about 4 grains, and tartaric acid about 20 grains. One to a pint = 1:2000. This formula, which is Laplace's, may of course be imitated by any apothecary and put in powder form in waxed paper. It has very great advantages over other formulæ.

---

***Leprosy Cured by Unna's Method.***—Dr. Dreckmann, of Vienenburg, reports a case of leprosy cured by treatment in the following manner:

The lower part of both legs and both feet, as well as both forearms and hands, are daubed twice a day with 10 per centum pyrogallic ointment, the remainder of the body with 10 per centum chrysarobine ointment by means of a stiff bristle-pencil or of a tooth-brush. The face, with the exception of the lower jaw region, is covered once a day with strong salicyl-creosote plastermull, daubing the latter with zinc-glue. This process, I may say, is the soul of the whole treatment, and has remained the same in its essential features from beginning to end, although with various modifications and experiments. To mention the most important among these: Experiments were tried with stronger ointments, pyrogallol up to 15 per centum, chrysarobine even as high as 25 per centum ointments. With pyrogallol, the experiment was punished almost regularly by a pyrogallol intoxication after 2 or 3 applications of the ointment; chrysarobine, which was decidedly less noxious, only induced an increased irritability of the skin. In both cases, the treatment was interrupted for 1–2 days, to subject these conditions to the proper treatment.

If it is desired to increase the intensity of the treatment, Unna's plastermulls are to be recommended. Many nodosities, especially on the back of the hand, which remain almost unaltered by the ointments, disappeared after 2–3 applications of the plaster.

A nodule in the lobe of the ear, having the size of a pea, and doubtless of leprous nature, vanished forever in one night before the chrysarobine plaster, in which I enveloped the whole concha. Being surprised myself by this result, I left this ear without any further treatment, in order to see whether the nodule might not return in one form or another. There was never any new disturbance.

In fact, I have succeeded in removing definitely the most obstinate indurations of the skin by means of plaster. Pyrogallol plasters are preferable to chrysarobine plasters with regard to efficiency.

Single nodules in a favorable location which allows to encircle them with the fingers, may simply be taken out with the knife; the wounds, caused by the operation, heal exceedingly well, and I have never seen in any place a reappearance of the induration. For I will not omit to mention that it happened to me several times that a nodule, which I had covered with a plaster, had disappeared from that very place on the following day, but only to be located near by, although in a more enlarged and mollified form. For this reason, general treatment should never be suspended, as long as there is one single nodule to be found somewhere.

Special attention should be bestowed on the treatment of the face. The well known Facies leonina, with its sombre, repulsive, fear inspiring expression has to be improved by all means, not only because it is a morbid condition, but also, because the patients themselves attach considerable importance to this improvement which saves them from being immediately recognized as lepers. For this purpose, I have not only removed the existing indurations by means of ichthyol and chrysarobine plasters, but I have radically detached the skin more than a dozen times by means of salicyl-creosote plasters, daubed with zinc-glue. For the purpose of finally rendering the complexion as nearly as possible similar to that of healthy persons, Unna recommends ichthyol ointments as the most efficient remedy.

As for the affections of the mucous membrane, the conjunctival catarrh disappeared by spontaneous action, doubtless in consequence of the general treatment. The ulcer in the nose, the induration on the tonsils as well as the little nodule on the epiglottis were removed or cured by cautery in 6 sittings. Treatment of the ulcers with nitrate of silver, boracic acid, did not succeed, nor emollition of the indurations on the tonsils. During the whole treatment, ichthyol was administered internally, doses being in the beginning 0.4 grams in pills, then after a fortnight, 1.0 pro die, by gradual increase. After that, I proceeded with larger doses of 2.0 grams, and soon of 3.0 grams pro die in capsules. I have never seen any unfavorable consequence of the internal use of ichthyol. As to the question whether ichthyol has really an appreciable action on the disease, when applied internally, I would not answer in a direct way. The fact is that the small doses of 0.4 pro die, applied in the beginning, were accompanied by as rapid an improvement in the condition of the patient, as later 3 grams pro die. I will not omit to state in this connection that Unna at present considers invigoration of the patient, especially removal of muscular debility and want of appetite, as the real value of internal administration of ichthyol.—*Pacific Record of Medicine and Surgery*, February, 1889.

---

***An Anomalous Form of Eczema.***—Dr. E. D. Mapother writes (*British Medical Journal*, January 5, 1889):

Last January I was consulted about a raw surface involving the right tragus, and the hairless skin in front of it, and at once its likeness to Paget's disease of the mammary areola struck me. It was oval, about 1½ inch vertically and an inch transversely, florid and moist as is the glans penis during balanitis. The patient was a married woman of 40, long troubled with uterine maladies. Refering to my notes, I found records of two very similar cases, unilateral and in females, but of diverse ages—45 and 12. In the latter there was also a small patch a little above the eyebrow. The uniform, florid, oozing surface without granulations, hard and slightly raised, but without the rolled over edge of rodent ulcer, without pain or much itching, stubbornness to treatment, without occasional disappearance, characterized all the cases. Dr. Crocker has observed a like condition on the scrotum.

Some physiological analogies group these regions: in all the sebaceous glands are very large; those round the nipple were, by Bidloo in 1685, described as supplementary mammary glands. The soakage of the skin by the overflow of milk or by the saliva of the infant may be prevented by their oily secretion, and in the scrotum and parotid region a similar waterproofing protection may be afforded against the urine and the sweat falling from the temple respectively. All agree as regions in which the arrival of puberty is manifested. In the female the scanty hair in front of the ear remains of the lanugo kind, and the glands are correspondingly large. The raw surfaces occasionally noticed round the lips, the nostrils, the eyelids, and round the anus are somewhat like, but they are rarely so chronic as Paget's disease, or the condition I note in the parotid region. All the cases healed, a slightly depressed unpigmented cicatrix remaining. Some forms of eczema undoubtedly leave scars, reaching well into the papillary layer. In 1879 Professor McCall Anderson saw with me a girl aged 4, who had somewhat similar raw eczematous patches on one cheek, and on the flexures of both knees and one elbow and ankle. The oozing was excessive, and did not cease for six years after, when rickets of the spine appeared, due probably to the saline flux. Pale, somewhat concave scars endure.

It may be mentioned that Paget's disease of the mammary areola appears to be rare out of England; so say Scotch and American observers; and except a case which I observed in St. Vincent's Hospital, I can find no record of an instance in Dublin. My patient was an Englishwoman. It may be that there is a peculiar microbe which has not been freely imported, nor has found a fit soil. The curative effect of that greatest of parasiticides, mercury, in the cases here noted, supports that probability, as also does the apparent usefulness of exclusion of air. All forms of eczema on the face are obstinate, because of the unavoidable access of light and air. The earlier cases I treated by an ointment containing carbolic acid, mercurial ointment, and vaseline; in the latest, that most valuable natural agent, lanolin, was employed as the vehicle; but in all, the surface being so limited, exclusion of air was attained by Seabury and Johnson's rubber plaster carefully adjusted.

---

***Prevention of Summer Diarrhœa in Children.***—DR. L. EMMETT HOLT says (*Medical News*, Feb. 23, 1889):

The treatment of follicular ulceration of the intestine is extremely unsatisfactory. I believe that the great majority of these cases are fatal. Certainly, I have never seen at autopsy in a child anything which resembled a cicatrized follicular ulcer.

Successful treatment must be in the nature of prevention.

Prevention must have regard to all the milder intestinal catarrhs.

Regarding neglected diarrhœas during dentition, so much has been said recently that it is scarcely necessary to enter here again a protest. There is to my mind no more reason why an intestinal catarrh should not be treated, and, if possible, cured during dentition than at any other time. The fact that a child with whooping-cough is extremely liable to bronchitis and pneumonia has never been given as a reason why these complications should not be treated promptly and energetically when they arise.

Is an intestinal catarrh ever salutary? This is questionable. A number of loose movements may be of advantage to expel undigested food or other irritating materials from the intestine, but that a persistent intestinal catarrh, even if not severe, is an advantage to any child at any period remains to be proven.

The medical profession should take strong ground against the prevalent popular opinion, that so long as the general health is not affected, an intestinal catarrh is not only of no importance, but may, during bronchitis or dentition, even be beneficial, and that to cure it might be injurious.

It is in such cases as these that though amenable to proper treatment in the earlier stages, when allowed to run on, as they often are for weeks or even months, the foundation for grave and even fatal forms of diarrhœal disease is often laid.

The prophylactic treatment involves then the early recognition and intelligent treatment of all the forms of dyspeptic catarrh; in other words, it means that we must secure proper digestion, and this depends chiefly upon proper feeding.

Our attention has been repeatedly called of late to the importance of seeing that our milk and other infant foods are pure and free from germs and putrefactive products. This is all important. Another danger which has not been often enough emphasized is overfeeding.

During the past two years I have been trying to get at some exact data regarding the proper amount of food which an infant, who is artificially fed, should receive at the different periods. This has been studied, first, by measuring carefully at autopsies the capacity of the stomach; and, secondly, by weighing healthy infants who were nursed at proper intervals, before and after they were put to the breast.

While I have not yet accumulated sufficient statistics for publication, still enough has been learned so far to show that the figures given in most of our books are altogether too large, and that the vast majority of hand-fed infants are *very greatly overfed.*

Difficulty and failure may result from this fact where every other condition for success has been attended to.

In conclusion I would emphasize the following points:

1. Children should not be overfed at any time, but especially not in summer.

2. At this season, also, every dyspeptic catarrh should be attended to; many of these are promptly curable by merely clearing out the intestine and then cutting down the quantity of food.

3. Should an intestinal catarrh, even a very mild one, continue for two or three weeks, one may be pretty certain that he has something more than a functional disorder to deal with.

4. Every mild catarrh should be looked upon as the possible precursor of a severe type of intestinal disease, either near or remote.

5. In the treatment of all diarrhœal diseases it should be borne in mind that there is something more to be considered than the bacteria and the products of decomposition, viz., the anatomical changes.

---

*Alum in Bread.*—Professor J. W. Mallet, of the University of Virginia, has been pursuing an interesting course of investigations into the effects produced by the use of alum in bread, and has found that, as has long been assumed, it is injurious. In the United States the greater part of the baking powders sold, it has been found, are made with alum, the acid phosphate of calcium, bicarbonate of soda and starch. The result of Professor Mallet's inquiry, as given in the *Pharmaceutical Journal,* has been to show that these powders give off very varying proportions of carbonic acid gas, and therefore different proportions have to be used for the same quantity of flour to produce the requisite porosity in bread. When moistened with water they yield small quantities of aluminium and calcium salts in a soluble form. Most of them leave, after use, the greater part of their alumina in the form of phosphate; but when acid phosphate of calcium is not used alumina is left. As the baking temperature in the interior of bread does not exceed 212° F., neither the water of combination of alumina or of its phosphate is removed from the residues of baking powder so used. However, in doses not very greatly exceeding such quantities as may be derived from bread as commonly used, Professor Mallet has found that hydrate and phosphate of alumina produced an inhibitory effect upon gastric digestion. He considers that this effect is probably a consequence of the union of alumina with the acid of the gastric juice, and at the same time of the precipitation of the organic peptic ferment in an insoluble condition like a kind of lake. A similar action may also be exerted by hydrate of alumina upon some of the organic matters of food. From the general nature of the results obtained, it is inferred that not only alum itself is injurious, but that likewise the residues resulting from its use in breadmaking must be ranked as objectionable, and that the practice of adding alum should be studiously avoided when the object aimed at is to make wholesome bread.—*The British Medical Journal.*

---

***Tape-Worm in an Infant on Raw Meat Diet.***—Dr. J. A. Shaw-Mackenzie relates (*British Medical Journal,* Jan. 5, 1889) the following case:

A. B., aged 1 year and 7 months, was the third child of healthy parents; it was weaned at 9 months, and fed on milk and broth, etc. It was a well-developed child, but always suffered from constipation. In May last, at 12 months old, after a severe attack of bronchitis and laryngitis, raw meat diet was begun, an ounce to an ounce and a half of finely scraped and pounded beef being given on alternate days. The child improved greatly, but in October began to fall off in looks; and on the 21st joints of tape-worm were noticed in the motions. Two fifteen-drop doses of liquid extract of male fern brought away several feet of the worm, but though small proglottides were noticed, no head was found, and though a repeated dosing was gone through a fortnight later nothing has been noticed since.

*Remarks.*—The possibility of tape-worm (tænia mediocanellata) following a raw or underdone beef dietary is well-known, but I do not think it is generally realized that the "raw meat diet" of infants carries with it the above disadvantageous chance.. During three years as house-surgeon and registrar at the Victoria Hospital for Children and in private I have never met with a case, and it would be interesting to know (with the now more general raw meat dietary) whether tape-worm in children is more common. The early addition of meat to a child's dietary is opposed to popular ideas, but the advantage, especially to the weakly and delicate, is so obvious by their rapid improvement and development that it seems disappointing to find that there is an "element of truth in the popular prejudice" of mothers against it. I am sure that children brought up on meat diet are very different in health, spirits, and physique from children brought up on a farinaceous diet, but in future I would recommend that all pounded meat should be first scalded with a little boiling water or beef tea, and I shall be inclined to push raw chicken diet, which from experience I have found children take readily and improve upon, with no apparent disadvantage.

---

***Nature and Treatment of Diabetic Coma.***—Dr. Stadelmann, of Dorpat, in a recent article in the *St. Petersburgishe Wochenschrift,* points out the great similarity which seems to exist between the coma of diabetes and the condition produced in herbivorous animals by inducing acid intoxication. Amongst other points, he refers to some analyses by Minkowski, of the gaseous contents of the blood. In the normal condition, the blood of the rabbit contains 25 per cent. of carbonic acid; but when the animal is suffering from artificially induced acid intoxication, the carbonic acid is diminished. Thus in one instance Minkowski found it 16·4 per cent. with a moderate degree of intoxication; when the latter was increased, the percentage of carbonic acid fell further, first to 8·8 and finally to 2·9 per cent. In order to compare this with the gaseous changes in the blood of diabetics, he examined the blood of a patient before and during coma, the carbonic acid being respectively 17·0 and 3·34 per cent. In order to ascertain whether this diminution of the carbonic acid in the blood was merely due to coma as such without reference to its cause, he examined the blood of a comatose patient, not a diabetic, whose condition was due to meningitis. Here the carbonic acid amounted to 28·2 per cent. The acid existing in diabetes appears to be oxybutyric acid, which in some cases appears in the urine to the extent of something like three ounces per diem. Some years ago Dr. Stadelmann found a new acid, which he believed to be crotonic acid, in considerable quantity in certain cases. He now, however, considers it merely a substitution product from oxybutyric acid. The indications for treatment supplied by these views are of course to combat the acid by large quantities of alkali. Several attempts have been made to treat diabetic coma by injecting into the veins from one to four ounces of carbonate of soda dissolved in about four pints of water, with a little chloride of sodium. In only one instance, however, has this proved successful, and unless it is done very early no good result can be fairly expected of it. It is found that the urine in twelve hours after the injection is intensely acid. Better results are to be obtained in attempting to ward off coma by giving alkalies freely. Thus Dr. Stadelmann prescribes about an ounce of tartrate or citrate of soda dissolved in about half a pint of soda water two or three times a day, and has found great reason to be satisfied with this line of treatment. Of course, if coma should come on, he would have recourse to alkaline intravenous injections without loss of time.—*The Lancet*, Feb. 9, 1889.

---

***Opiates in Diabetes Mellitus.***—In a paper on the relative value of opium, morphia and codeia in diabetes mellitus, read before the Section of Pharmacology and Therapeutics of the British Medical Association, Dr. Thomas R. Fraser said that he had arrived at the following conclusions:

1. The case was one in which mere restriction of diet did not have so marked an effect as occurs in many cases. The prospects of successful treatment were not, therefore, very hopeful.

2. Codeine had a very decided effect in reducing the quantity of urine, sugar and urea. When contrasted with the reduction produced by restricted dietary alone, the addition of nine grains of codeine in the day lessened by about one-third, and of fifteen grains of codeine in the day by about one-half, the quantity of fluids drunk, and the quantity of urine, sugar and urea, and it slightly reduced the specific gravity of the urine.

3. The addition to fifteen grains of codeine of the one-twentieth, and afterwards of the one-tenth of a grain of sulphate of atropine, caused a still further, though not a large, reduction.

4. After the administration of codeine had been stopped, an interval of six days on restricted diet, without any medicinal treatment, was not sufficient for a deterioration to occur to the conditions present before codeine had been given.

5. The subsequent administration of half a grain of opium thrice daily produced a considerable reduction. With one grain of opium thrice daily the reduction was to less than one-half, when contrasted with the amounts during a restricted diet alone, and before any medicinal treatment had been adopted. One grain and a half thrice daily produced a further reduction; and when to it was added one-twentieth of a grain daily of sulphate of atropine, a still further reduction occurred.

6. Restricted diet, with one third of a grain of hydrochlorate of morphine thrice daily, or one grain daily, also produced a marked reduction; and the conditions relative to the points under investigation were even more satisfactory than when fifteen grains daily of codeine were being administered. While this small quantity of morphine was being taken, the fluids drunk by the patient were only one-third, the urine and sugar less than one-half, and the urea about one-half of the amounts during the period of restricted diet alone, before medicinal treatment had been commenced.—*Northwestern Lancet.*

---

***The Bacteriology of Puerperal Fever.***—CZERNIEWSKI (*Arch. f. Gyn.*, B. xxxiii., H. I.,) in an elaborate paper discusses some of the unsettled questions of puerperal bacteriology. Many authors are of the opinion that the streptococcus is the specific microbe of puerperal fever, at least that which pertains to the severer forms of fever (Pasteur, Lomer, Gönner). Of the cause of the lighter forms there is as yet no settled opinion. Fritsch makes two forms of fever—septic or severe and non-septic or light, believing that the septic fever may be produced by non-pathogenic organisms. Olshausen separates the non-malignant (peri- or parametritic) from the septic variety without attempting to explain the difference in the pathogeny. Charpentier recognizes a traumatic and a septic fever. Schroder says putrefaction of lochia may occur from access of atmospheric germs. Zweifel does not commit himself on the question whether the lighter varieties of puerperal fever are of septic origin or not. The severer form he ascribes to the chain cocci. He apparently thinks the chain cocci may in certain cases cause only the milder grades of fever. The larger proportion of the lighter forms are produced by the absorption of putrefactive products (ptomaines, sepsin).

Charles appears to be of the same opinion as Charpentier. So far as the writer knows Credé is the only authority who takes a decided stand on the theory that all grades of childbed fever are due to infection with one and the same poison. The different results of infection he attributes to differences in the resisting power of the organism, the sites of invasion and channels of diffusion. If the peccant germs expend their force upon the tissues, the lighter forms of fever result; if they gain access to the blood or lymph-vessels, the graver forms are produced.

Rosenback, Passet, Norden, Mejerowitz, Biondi, and others declare the identity of the pyogenic streptococcus and that of erysipelas. Pawlowsky, Hoffa, Winckel believe them to be different organisms.

Dr. C. has studied the subject by the aid of an elaborate series of bacteriological investigations conducted in the lying-in hospital at St. Petersburg. He examined the lochia of sick and healthy puerperæ and the fluids from the cavities of bodies dead of puerperal fever, and made cultures therefrom. His conclusions are as follows:

1. Micro-organisms are very seldom found in the uterine cavity or the lochia of healthy puerperæ.

2. The lochia of healthy lying-in women possesses neither phlogogenic nor pyogenic properties.

3. In most cases of light puerperal fever the streptococcus may be found in the uterine cavity and the lochia.

4. In fatal cases of childbed fever (septicæmia, lymphatic form), the streptococcus is found in the lochia, and after death in all the organs and fluids of the body.

5. The streptococci of the light and of the fatal forms of fevers are one and the same organism.

6. The streptococcus of puerperal fever by inoculation in animals is capable of producing abscess or erysipelas.

7. In sick puerperæ the streptococcus gives rise to a degeneration of the parenchymatous organs, hyperæmia of the serosa, and to more or less exudation.—*Brooklyn Medical Journal*, March, 1889.

---

***Hydroxylamin in Skin Diseases.***—Dr. Eichoff, of the Municipal Hospital, Elberfeld, has found an admirable substitute for pyrogallic acid, chrysarobin, and other powerful reducing agents used in external applications for skin diseases in hydroxylamin, which is, chemically speaking, an ammonia in which one of the atoms of H is replaced by HO. The most suitable compound for dermatological use is the chloride, the formula of which is $NH_2OH.Cl$. This occurs in colorless, strongly hygroscopic crystals, which are readily soluble in water, glycerine or spirit, the solution showing an acid reaction. When introduced into the blood, hydroxylamin forms methæmoglobin, the blood rapidly becoming of a deep brown color. In large doses—that is to say, 0·01 gramme per kilogramme of body weight—it produces hæmaturia in consequence of the destruction of the red corpuscles. It also acts on the nervous centers, producing narcosis. The high reducing power possessed by hydroxylamin renders it a powerful poison to low organic forms, and on this acccount it is to be very strongly recommended in dermatology. The preparation used by Dr. Eichoff is the hydrochlorate dissolved in a mixture of equal parts of glycerine and spirits of wine in the proportion of 1 per 1000. This is applied with a brush to the affected parts of the skin, which must first be carefully washed with soap three to five times a day. In this way he has treated five cases of lupus, five of ringworm, and one of parasitic sycosis, with excellent results. These were specially remarkable in two cases of very severe lupus. Dr. Eichoff is hopeful that this remedy, which may sometimes perhaps be applied in the form of subcutaneous injections, may be found useful in psoriasis, parasitic eczema, and even in lepra and syphilis. He, however, warns those who propose to try it that it is a very powerful irritant, and that even for outward application a strength of 1 per 1000 is quite enough.—*The Lancet*, Feb. 9, 1889.

---

***Surgical Shock.***—Upon this most important topic Mr. Christopher Heath says: While many surgeons, myself among the number, rarely employ the carbolic spray nowadays, yet even without this there is, I believe, considerably increased risk to patients in the exaggerated slowness of modern surgery. For a patient to breathe the vapor of ether or chloroform for over an hour is a serious matter for both heart and lungs; and when we add to that a steady drain of blood going on for an equal time, one cannot be surprised at the production of deadly shock. And is there any gain after all in elaborate dissections of the living as though it were a dead body? I believe not; and, having had a fair amount of experience in removing tumors of all sizes from all parts of the body, I would urge surgeons to use the left hand a little more and the right a little less than now seems to be the fashion. I mean, to make forcible traction with the one hand whilst avoiding the tense structures with the other, so as to avoid dissecting-room proceedings with scalpel and forceps as much as might be. I hope I shall not be misunderstood as decrying careful dissection in appropriate cases—for example, the ligature of large arteries; for I fear, from what I see in examining candidates for the higher surgical degrees, that a blunt director must be used by some teachers in what I consider to be a very dangerous manner. The following guards against shock are strongly recommended: a rectal injection of brandy and hot water prior to and a subcutaneous injection of morphine and atropine after an operation.—*British Medical Journal.*

---

**Phenacetine.**—Apparently one of the best of the modern antipyretics is a substance described by Hinsberg and Kast as para-acetphenetidin, a substance analogous as regards its chemical constitution to antifebrin. We have already a number of times alluded to the properties of this substance (*Therapeutic Gazette*, 1888, pages 43, 142, 699), and although the testimony as to the action of this preparation as an antipyretic and antineuralgic appears to be unanimous as to its value and freedom from danger, it has attracted no attention among English-speaking members of the profession. This preparation, phenacetine, as first prepared, was a reddish, odorless powder, insoluble in water and in glycerine, and thoroughly soluble in hot alcohol and alkaline liquids. It has been recently prepared in colorless, crystalline needles, which are claimed to be soluble in acetic and lactic acids and in hot oils. Extended experiments on dogs and rabbits have shown that it is almost inert in doses of from 15 to 30 grains given daily for days at a time. When the dose is increased up to 45 and 75 grains in large dogs it produces accelerated respiration, sleepiness, disturbed co-ordination, and vomiting, and, in still larger doses, methæmoglobin is produced, as in antifebrin-poisoning. Even after this symptom has appeared, however, recovery has almost invariably occurred. Dr. Hoppe (*Deutsche Medizinal Zeitung*, 1888, No. 92) has made a number of experiments on man, administering doses of from 15 to 40 grains, and has found that after a time the system becomes accustomed to the remedy. The only disagreeable effects produced by these amounts were sleepiness, dizziness, nausea, and slight chilliness, the temperature, as consequent on doses of 30 grains, being only reduced a few tenths of a degree Centigrade. It appears, therefore, to be almost free from toxic properties, while his experiments made in the Jewish Hospital in Berlin have confirmed the result of the experiments as already published by other observers. It has proved absolutely harmless, the only disagreeable after-effect being profuse perspiration, ringing in the ears, followed by weakness, and only in individuals already depressed by disease. As an antipyretic in children, doses of about 2 grains reduce the temperature from 1° to 2° C., a single large dose producing more effect than repeated small doses.

Led by the analogy of phenacetine in composition to antipyrin and antifebrin, Dr. Hoppe has likewise tested the properties of this drug in twenty-five different forms of neuralgia, in the majority of which relief of pain followed its employment. Various cases of headache were also relieved by its use within half an hour to an hour; and he believes that in neuralgia, as in febrile disease, it is equally as efficacious as antipyrin, and is preferable to it on account of its freedom from danger.

Dr. Rumpf (*Berl. Klin. Wochenschr.*, No. 23, 1888) likewise experimented to a considerable extent with phenacetine as an antipyretic and in the treatment of neuralgia. Dr. Rumpf believes that as an antipyretic it is as active as any yet introduced, since he has found that a single dose of 15 grains given to adults may reduce the temperature in the febrile state from two to three degrees in two hours; even half the dose has produced the same effect with no disagreeable complications. In eight cases of hemicrania doses of 15 grains produced great relief, while in seven cases of neuralgia of different nerves it has likewise been very satisfactory. Dr. Rumpf describes phenacetine as a drug which, in doses of 15 grains to adults or 3 grains to children, is an absolutely safe, reliable, and satisfactory antipyretic; while in doses of 15 grains it is highly recommended as an anti-neuralgic remedy in all cases of vasomotor neuroses, in the lancinating pains of tabes and the neuralgias of chronic neuritis.

Drs. Misrachi and Rifat (*Bulletin Gén. de Thérapeutique*, June 22, 1888) have confirmed in all respects the statements which we have already published as to the action of this remedy. It is these authors who have determined the solubility of phenacetine in lactic acid. They have found that this solution is not disturbed by the addition of water as long as the temperature does not fall below 33° C. It is evident that this discovery is a great addition to the practical value of this remedy, since its high insolubility has been the principal objection to its use in therapeutics. After the administration of the lactic-acid solution it is rapidly absorbed, and has been capable of detection in the urine.—*Therapeutic Gazette*, March 15, 1889.

---

***Germicides in Typhoid Fever.***—Although in typhoid fever there is a better opportunity for using germicides in sufficient concentration to render them effectual, practitioners appear to be slow in adopting this idea in its treatment. This is doubtless in great part due to the very fair results obtained by the treatment already in vogue. The antiseptic methods practiced in the great German hospitals, and the expectant plan pursued in this country, have yielded a percentage of recoveries which is certainly remarkable when we consider the serious nature of the disease, and the dangerous complications and sequelæ which beset it. But no result is good enough if there be room for a better.

Another reason for the slowness of the profession to take up the intestinal germicides has undoubtedly been the thought that, while the intestinal canal has served for the introduction of the disease-germs into the body, the symptoms are produced by the penetration of these germs into the blood-vessels, and their passage to all parts of the body. To what extent and at what stage of the disease this takes place, we are not as yet informed. Indeed, we are in want of a series of examinations of the tissues and fluids of the body, during the progress of typhoid fever, with a view of determining this interesting point. Judging from the effect of germicidal treatment, we are warranted by clinical observations in believing that the intestinal canal is at first the sole theater of the microbic operations, and that the general symptoms are produced by the absorption into the circulation of poisons generated in the intestines by the specific microbes of the disease; further, that there is a time when a microbic invasion of the blood really occurs; after which the exhibition of intestinal germicides fails to be followed by the marked benefit which accrues from their use previous to that time.

It may be noted that even before the promulgation of Klebs' discovery, the remedies which had obtained the esteem of clinicians in the treatment of this disease are those which possess some local germicidal properties. How else can we explain the claim of Wunderlich, that typhoid can sometimes be aborted by a few doses of calomel? Or that cases treated throughout with iodine give a better percentage of recoveries than those treated without it, all other conditions being the same? It is possible that nitrate of silver, the mineral acids, quinine and turpentine owe their reputation in great part to their local germicidal effects.

During the past year there have been several important publications upon this subject; especially noteworthy as showing that several observers, working with different agents, have obtained very favorable results.

Gramshaw[1] reported a series of 116 cases, treated with carbolic acid and iodine, without a single death.

Bouchard[2] makes use of naphthol and salicylate of bismuth, and claims results more favorable than were obtained from empiric medications, and the treatment of symptoms. As he associates with this treatment the use of baths, quinine, calomel, etc, it is not possible to judge definitively of the exact value of intestinal antisepsis in his very numerous successful cases.

To these observations is now added an interesting paper in *The Practitioner* by J. Mitchell Clarke, on beta-naphthol in enteric fever. He calls attention to the ease with which toxic bodies, found in the intestines, normally, or through the intervention of the disease, gain access to the organism through the intestinal ulcers. Bouchard showed that beta-naphthol is only toxic in doses of about eight ounces per day for an adult of average weight, while forty grains in the 24 hours suffice to keep the bowels aseptic. This is given in milk, or in gelatine capsules. Small doses frequently repeated are preferable.[3]

Acetanilide or phenacetine were also used when the fever exceeded 102° F.

In the five cases treated by naphthol throughout, the abdominal symptoms were favorably modified, but the fever showed its usual course, the highest range being 103.5° to 104.8°. In two cases the naphthol had to be stopped on account of gastric disturbance, excited by the drug. In one case a relapse occurred after defervescence from a mild first attack. Convalescence was unusually rapid in this series of cases, and the patients were less reduced in strength than usual.

His conclusions are:

1. That the production of an intestinal antisepsis is a rational mode of treating enteric fever, and that beta-naphthol is a safe and tolerably efficient agent for this end.

2. That by its use in the above cases the duration of the disease was shortened, and the intensity of the symptoms directly arising from profound disturbance in the alimentary canal was lessened.

3. That the tendency to the occurrence of splenic enlargement, albuminuria and secondary complications, such as boils, abscesses, etc., of purulent infective origin, is diminished.

4. That complete convalescence is more speedily and satisfactorily attained, and that there is less risk of a propagation of the disease to others.

In June last the writer presented a series of twelve cases treated with the sulpho-carbolate of zinc. Eleven of these were thus treated from the beginning, and all recovered. The other case was almost moribund when it came into the writer's hands, from repeated intestinal hemorrhages, and cannot be counted against the treatment, except as showing that there is a time when intestinal antisepsis ceases to be curative. Since that time twenty-two cases have been treated by the same agent, all of which recovered.

The sulpho-carbolate of sodium was substituted for the zinc salt in some instances, the results being apparently identical.

Neither Clarke's list of seven cases, nor our own of thirty-three, is sufficiently extensive to warrant positive deductions; but it may be of some interest to point out the differences in the action of the two agents used:

1. Intestinal antisepsis is procured by

---

[1] Philadelphia Medical Times, Aug. 1, 1888.

[2] La France Médicale, Jan. 3, 1889; page 3.

[3] The following is Clarke's formula:

| | | |
|---|---|---|
| R | Beta-naphthol | gr. xx |
| | Tinct. aurantii | f. ʒ ij |
| | Syr. limonis | f. ℥ ss |
| | Mucil. tragacanthæ | f. ℥ iij |
| | Aqua, q. s. ad | f. ℥ vj |

M. S. Dose, f. ℥j.

both, requiring 40 grains *per diem* of naphthol and 30 grains of the sulpho-carbolate (in adults).

2. Gastric disturbance resulted in two cases out of seven from the naphthol; in none from the zinc.

3. No decrease in the fever was obtained from the naphthol, while in every case the sulpho-carbolate caused a fall of at least one degree.

4. Dr. Clarke does not state that the cerebral symptoms were either moderated or prevented by naphthol; while this formed a marked feature in all our cases but one.

5. The tendency of secondary suppurations, probably resulting from the entrance of pyogenic bacteria through the intestinal ulcers, was diminished in both series.

6. We think that the effect on the intestinal symptoms was more marked in our own cases, though it may be simply that Dr. Clarke has not dwelt on this point.

Altogether, the advantage in every particular appears to be on the side of the sulpho-carbolates.—*Medical Times*, February 1, 1889.

---

***Subcutaneous Silk Ligature in Fracture of the Patella.***—At the meeting of the New York Surgical Society on January 9, Dr. Lewis A. Stinson showed two patients with recent fracture of the patella treated by subcutaneous silk ligature. During the preceding two months he had thus treated five patients with recent fracture, the two now presented being the first and third of the series. The second had been discharged cured; the fourth and fifth were still under treatment and doing well.

The method employed might be called a modification of the external silk ligature employed some years ago by Volkmann, and had been suggested and employed in the first two cases, under the speaker's supervision, by Dr. E. W. Clarke, house surgeon at the New York Hospital. It consisted in passing a silk ligature through punctures made in the skin, through the tendon of the quadriceps femoris and the ligamentum patellæ and under the skin in front of the patella, in such a manner that by drawing the ligature tight the two fragments of bone were drawn together. He employed a long half-curved Hagedorn's needle and a stout braided silk ligature. The four punctures (through the skin and the sides of the tendons) were made close above and below the patella—at its four corners, so to speak. The needle was entered at the lower right-hand corner, carried through the ligamentum patellæ to the bone, then across and out through the opposite puncture. This made a loop through the lower tendon. Then the needle was entered beneath the skin at its recent point of exit and carried upward between the skin and the front of the patella and brought out at the upper puncture on the same side. It then reëntered at this upper left-hand puncture through the tendon of the quadriceps, crossed beneath it, emerged at the opposite puncture, and thus made a loop through the upper tendon. The needle was then passed in at this fourth puncture (through the skin) and carried down under the skin and in front of the patella to emerge at the first puncture (where it had entered). The two fragments of the patella were then pressed into close apposition, the silk cord was drawn tight and tied, and its ends were cut short. The entire suture being thus subcutaneous, the punctures through the skin were closed with a single suture passed through each. It was not necessary that the ligature should include the entire width of the tendon, but it was sufficient if it embraced just enough to give it a firm hold. It should also be passed close to the attachment of the tendon to the bone, in order that it might not become slack by cutting through the tendon toward the bone, under the strain. The silk-worm gut was apt to break at sharp turns. Catgut became absorbed too soon unless treated with chromic acid. The union obtained was not bony, but a close fibrous union; however, this had been shown to be true of the union of the patella in most instances where examination had been made after death, even when the fragments had been wired together. He had in a single instance produced bony union after uniting the fragments with catgut. He applied only a simple posterior splint. The stiffness of the knee which patients at first experienced he was convinced was due to contraction of the internal and lateral ligaments of the joint and soon disappeared when the use of the limb was resumed.

In one case ten days had elapsed after the accident before the operation was done; in the others only a few hours. In the first case the needle had broken and a transverse incision had been made to remove the broken end. Silk-worm gut had been used in this case for a ligature, and its manipulation had been faulty. Twelve days later the fragments had been found to be an inch apart, probably owing to kinks forming in the ligature when first introduced. The operation had been repeated, this time silk being used. Suppuration had followed, but the process had remained extra-articular. After a month the silk suture had been removed through the sinus, and the silk-worm gut suture two weeks later. Since then the openings had closed rapidly. The patient could flex the knee nearly to a right angle and no separation could be felt.

In the second patient the fracture had been close to the upper border of the patella, and the patient had been discharged well in about six weeks. In the third patient (presented) the fracture had occurred five weeks before; he was now able to flex the knee to a right angle, and no separation of the fragments could be recognized. The fourth patient had been under treatment three weeks; there had been no inflammatory reaction, and the fragments were not separated. In the fifth case only two days had elapsed since the operation.

The speaker thought the method was to be recommended, not only because of its own merits, but also because it was an efficient and apparently much less dangerous substitute for open arthrotomy and metallic suture—a method which, although almost entirely abandoned in Europe, was still having numerous victims in America. He knew of three cases, of which no report had been made, that had occurred during the past summer in the hospitals of New York where, after that operation, the joint had suppurated, amputation had become necessary, and eventually the patients had died.

Dr. J. D. Bryant asked whether the method would be available where there was marked separation of the fragments with difficulty in making coaptation, or when there was an interposition of fibrous tissue, or when a firm blood-clot filled the gap.

Dr. G. A. Peters suggested, in answer to Dr. Bryant's first question, that four, six, or even eight separate loops could be passed and drawn together.—*The New York Medical Journal*, March 9, 1889.

---

***Lead-Poisoning from Service Pipes, in Relation to Sterility and Abortion.***—Dr. Alfred Swann says (*British Medical Journal*, Feb. 16, 1889):

The following cases are of interest, inasmuch as they suggest, if nothing more, a possible danger arising from the supply of water through leaden piping for domestic purposes.

Case I.—Mrs. T., aged about 32 years, was confined in 1882. Since then she has had progressive ill-health, with constipation, digestive disturbances, frequent colic, rheumatic pains, and irregular menstruation, with now and then severe menstrual hæmorrhage. After cessation of menses for eight weeks an abortion occurred on March 17th, 1884. After cessation of menses for seven weeks, another abortion took place on September 10th, 1884.

Colic continued to be frequent, and severe constipation remained obstinate, and recurrent attacks of metrorrhagia became alarming. I had for a long time been of opinion that the water-supply, which came through lead piping of some length, was the cause of all the foregoing symptoms, and in the early part of 1887, my patient having been confined to bed for some months, iron piping was substituted. After this there was only one attack of colic, and no severe hæmorrhage; the bowels became regular, and menstruation became normal for a time. Pregnancy ensued, and my patient was confined of a healthy child on March 2nd, 1888. Since then she has remained in good health.

Case II.—Mrs. C., aged about 30 years, has had one child some seven years ago, in 1882. She came under my care in 1885, suffering from constipation, indigestion and colic. She has irregular and frequently profuse menstruation. She aborted after suspecting pregnancy for eight weeks, on January 6th, 1886. She has a blue line on the gums. I ascribed her abortion and previous illness to lead piping, and these were removed and iron pipes substituted. Colic became less severe and disappeared; the bowels became regular; menstruation became normal; then pregnancy ensued. On October 22nd, 1887, she was confined of a healthy child, and has since remained in good health. In the same house the servant suffered from rheumatic pains, colic, and constipation, until the removal of the lead piping, after which she regained her wonted health.

Case III.—Mrs. F., aged about 30 years, has two children. The last was born on September 7th, 1884. After this her health became seriously impaired; she had severe digestive disturbances, rheumatic pains in the limbs and loins, constipation, colic, and very irregular menstruation. Her life became a burden to her, no treatment being of any avail. I told her husband that I believed all her symptoms were due to the water-supply, but he ridiculed the idea. I referred him to Cases I and II. Even after receiving their evidence, his skepticism remained, but he removed the lead piping, and substituted iron. The result was as remarkable as in Cases I and II; all the symptoms disappeared and menstruation became regular. Then pregnancy ensued, and on May 11th 1887, my patient was delivered of a healthy child, and has since remained in good health.

I do not record these cases in a dogmatic spirit, nor do I aspire to scientific accuracy in my deductions from them, but there can be little doubt that in Cases I and II the cause of abortion and hæmorrhage was water contamination by lead, and in Case III one may reasonably suspect lead as being the cause of all the symptoms.

It has fallen to my lot to observe many cases of plumbism, and its relation to sterility and abortion only touches the very fringe of a vast subject. The influence of lead on the nervous, vascular, muscular, lymphatic, and digestive systems merits greater consideration than has hitherto been devoted to it.

To suppose that plumbism means only wrist-drop and paralysis of the extensors of the forearms seems to me to be illogical. I believe that in plumbism, neuritis is not confined to any particular set of nerves. Is not lead colic due to paralysis of the nerves regulating the muscular coats of the intestines? What is the meaning in cases of lead-poisoning of the tense pulse, the liability to epileptiform seizure, to cerebral and other hæmorrhages, to gout and uric acid, and to albuminuria and rheumatic pains? Surely these point to both nervous, vascular, and metabolic derangements which open up a wide field of inquiry for those who are interested in our food and water supply, and in public health generally.

This paper is written only to suggest grave possibilities to those who at present barely recognize a serious source of danger, and although I personally am satisfied of the far-reaching and lethal influences of lead in relation to many conditions not generally regarded as having any connection with it, yet I would prefer that those with more opportunities at their disposal than I possess would follow out a line of investigation which I have merely indicated. I am satisfied that they will find a fertile field for research.

---

***Methyl Chloride for Local Anæsthesia.***—Dr. B. Ward Richardson says: When chloride of methyl is condensed, it produces, on being liberated through a jet, an intense cold. It has therefore been used commercially for refrigerating the carcasses of dead animals intended for consumption as food. This use of it has led to the suggestion of employing it for freezing parts of the living body in the same manner as ether spray is employed for local anæsthesia. The idea of such use occurred to me soon after I introduced ether spray, but was abandoned because of the difficulty of managing the gas for the purpose required. The difficulty lies in so regulating the escape of the gas, as to be sure of doing enough without doing too much.

In itself the chloride of methyl has no local anæsthetic properties, as some ignorant notices of it in the general press would lead the reader to believe. It acts simply and solely like ether spray, by the cold it induces, and the surgeon who would employ it should fully understand that he is simply substituting one cold producer for another. As a matter of practice, ether or rhigolene spray for benumbing with cold is in every sense the readier and more practical method, and the principle at work is the same.

For causing local anæsthesia by extreme cold compressed nitrous oxide, carbonic acid, or sulphurous acid act in a similar manner to compressed methyl chloride. I once pursued a line of enquiry for local anæsthesia with nitrous oxide and with carbonic acid, the jet of each of which produced local anæsthesia. A jet of the nitrous gas playing upon the skin destroyed sensibility immediately by the cold it caused, and once acted with such intensity that a burn was the result. A workman, who was engaged in fitting up a nitrous oxide apparatus in my laboratory, brought his arm accidentally across a jet of the gas escaping at great pressure, and was so severely "chill-burned" that I had to treat him as for a burn from heat. This man told me that the accident was not uncommon amongst his class of workmen, and that they called the wounds that followed exposure "*chill burns.*"

After seeing the action of these gases as above described, it occurred to me that they might be used as cauteries for the removal from the skin of warts and small pendulous growths. Their jet is much less alarming than either the heated wire or the knife. It is also very rapid in its action, and at the moment painless, although, as a matter of course, there is some pain and soreness afterwards.

Here is a new line of practice for surgeons, for removal of foreign growths by *the freezing cautery*, a practice which I do not for a moment doubt they will one day take up and follow out to important results. There are other gases beside those named which might be used. Compressed chlorine, delivered in a very fine jet, would be magically destructive. Combinations of true anæsthetics and freezing jets might also easily be obtained, under which large portions of the body could be painlessly removed.

In commencing this line of practice there can be no doubt as to the best gas to commence with. The best gas is carbonic acid. It is cheaply and readily made; it is easily condensed, admitting even of being condensed into the solid form, but on being set free as spray from the liquid state it causes sufficient refrigeration. One great advantage of it is that, unlike methyl chloride, it is not inflammable, and can be used, therefore, with artificial light. It is also slightly anæsthetic.—*The Asclepiad*, Vol. VI, No. 21.

---

***Chronic Headaches of Functional Origin.***—In an article on this subject Dr. Charles L. Dana says in regard to treatment:

The *treatment* of *headaches* of *young children*, for example, brings us into an almost special line of cases. In the city of New York at least, these headaches are best treated as a rule by giving small doses of the iodide of iron or the citrate of iron and quinine. In school children headaches have often to be treated by removal from school, the use of tonics, change of diet, and the application of glasses suitable to any eye-defects that may be present. But glasses should be the last thing tried, unless the visual trouble is very marked. In some children arsenic acts well.

As to the *therapeutics of headaches of adults:* Headaches among brain-workers require, as a rule, a different class of remedies to those among muscle workers. In the former class nervines like antipyrin, caffein, the bromides, act well, while attention to diet, exercise, and the eyes is especially required. Among the laboring classes, especially women, anæmia, malaria, syphilis, and rheumatic influences must often be attended to. There are sexual differences also to be borne in mind. In my experience, for example, antipyrin has been much oftener successful in men than in women. This may, however, be only a coincidence.

I shall conclude the subject of therapeutics with a few remarks on symptomatic remedies. Among the best of these is muriate of ammonium in large doses, ℨss to ℨj, given in wafers. In the headache of neurasthenia, I have found that gr. v of menthol in hot water gives relief. In my wards at Bellevue Hospital a combination of menthol gr. v to x, and antifebrin gr. v to x, is often used. Phenacetin is also a good remedy. A practical point of importance in the use of antipyrin is the dosage. Often the best results are gotten by small doses frequently repeated. The much advertised effervescent preparations for headache contain too small a dose of caffein or of bromide to be of the best service.

Of local applications a spray or lotion of aconitia; sheet lint soaked in twenty per cent. solution of menthol and wrapped on the head; solutions of cyanide of potash after the method of Trousseau; Rithet's tobacco and quinine snuff, are some of the measures indicated.

Every one meets now and then with cases of headache of obscure origin, obstinate in character and intractable to every kind of treatment. The use of iodide of potassium and of the strong galvanic current and static electricity has been of service to me in some such cases. The possibility of a diffuse neuritis as a cause of the pain in certain of these cases should be borne in mind.—*Medical Progress*, March 16, 1889.

---

***Mental Depression and the Excretion of Uric Acid.***—In the *Practitioner*, November, 1888, Dr. Haig contributes an interesting paper on the relation between the excretion of uric acid and mental depression. The author has published his researches on the relation of certain forms of headache to the excretion of uric acid (*Med. Chir. Trans.*, vol. lxx.), and he now shows that the mental condition of many patients depends on the amount of uric acid in the blood; when the acid is in excess it produces marked depression or irritability of temper, when this excess passes off there is a feeling of exaltation and sense of well-being. Many suffer from a certain amount of mental depression and heaviness at that time of the day at which the excretion of uric acid is normally at its greatest, that is in the morning between breakfast and lunch, during the time the acidity of the urine is lowest. This corresponds in every way to the condition artificially produced by giving alkalies, which wash uric acid in excess into the blood, so inducing depression; by giving acids sufficient to counteract the alkalinity of the blood, the heaviness and depression will give place to feelings of well-being, mental clearness, and pleasure in living. When the blood is strongly alkaline, and in a condition to hold much uric acid in solution, all the symptoms of mental depression are present, and are more or less marked according to the amount of acid present, the excretion of uric acid in the urine being at the same time proportionally in excess of the average. When a dose of mineral acid has been taken the mental conditions clear up exactly in proportion as the uric acid is cleared out of the blood, and its excretion in the urine diminishes. When a dose of acid has been taken to cure a headache or a fit of mental depression it is extremely common for some amount of shooting pain in the joints to be present while the acid is driving the uric acid out of the blood. By reducing suddenly the alkalinity of the blood in this manner, it is very common for the uric acid in the tissues of the joints to be deposited in the joints instead of passing off in the blood. In the treatment of cases of mental depression, where it is evident that the excretion of uric acid plays the chief part, it is not always certain that a dose of acid taken now and then will produce a cure; the prevention of the excessive formation must be aimed at by regulating the diet. A large amount of butcher's meat should not be allowed, but should be replaced by fish, fowl, and milk, and stimulants are to be avoided. In many severe cases the author has met with great success by insisting on a diet of bread, butter, milk, potatoes, and a large quantity of fruit, continued for weeks or months. At the beginning of this treatment the washing out of the uric acid stores may be hastened by giving about 15 grains of salicylate of sodium three or four times a day, or in some cases a dose of 20 grains every night is sufficient. This subject is worthy of continued study, and should commend itself to all medical men.

---

*The Value of Inhalations in the Treatment of Lung Disease.*[1]—A limited number of experiments made by the author on phthisical patients tend to show that inhalations of iodine, whether vaporized by the steam or the hand-ball atomizer, have no deep penetration, inasmuch as the iodine cannot be detected in the urine after the inhalation. Turpentine, on the other hand, when inhaled, imparts its characteristic odor to the urine, thus showing that it is absorbed into the blood. This may explain its efficacy in checking hæmoptysis.

The conclusions are as follows:

1. That the success of inhalations as a mode of medication depends principally on the easy convertibility into gas or vapor of such substances as are clearly desirable for the purpose.

2. That, consequently, bodies which are volatilized at ordinary temperatures are more readily absorbed by the lungs than bodies which have to undergo combustion before conversion into gases.

3. That all moist inhalations, where steam, watery vapor, or spray is the vehicle of medication, are but slowly absorbed by the lungs, and enter the circulation in small quantities, and in some cases not at all, the slow rate of pulmonary absorption contrasting strongly with the rate of gastric absorption of the same medicines when swallowed, as proved by their detection in the urine.

4. That medicinal inhalations are more useful in diseased conditions of the pharynx, larynx, and larger bronchi than in those of the alveoli and lung parenchyma.

---

[1]C. Theodore Williams, Lancet, 1888, ii. p. 700.

5. That in pulmonary disease the antiseptic respirators, while they lessen cough, and reduce expectoration, exercise no lasting remedial influence on the diseased condition of the lungs, and often seriously interfere with the freedom of respiratory effort, which is so desirable in the treatment of such affections.—*Boston Medical and Surgical Journal.*

---

***Treatment of Nasal Affections.***—Dr. Cozzolino, of Naples, recommends for the various affections of the nasal passages the following compounds:

*For scrofulous rhinitis.*

℞ Sulpho-carbolate of zinc, grs. v.
Salicylate of bismuth, grs. lx.
Iodol, grs. vi.
Tannate of zinc, grs. xxx.
Powdered talc, grs. cl.
M. Use as a snuff.

*Chronic catarrhal rhinitis.*

℞ Powdered alum,
Borax, ää 30 grs.
Menthol, 3 grs.
Tannate of zinc,
Tannate of bismuth, ää 45 grs.
Lycopodium, ʒ ij.
M.

or

℞ Salicylate of zinc,
Tannate of bismuth, ää ʒ j.
Powdered borax, 30 grs.
Salol, 23 grs.
Powdered talc, ʒ ij.
M.

*Simple acute catarrhal rhinitis.*

℞ Chloride of ammonium, grs. 45.
Salicylate of sodium, grs. 30.
Chloride of potassium, grs. 45.
M. To be used as a snuff.

*Hyperœmic swelling of the nasal mucous membrane.*

A frequent cause of reflex disturbances.

℞ Glycerin,

Water, ää ʒ iv.

Alcohol (rectified), ʒ iss.

Menthol,

Cocaine, ää grs. 3.

M. Use three to four times daily.

*Very acute coryza.*

℞ Water,

Alcohol (rectified), ʒ iv.

Carbolic acid, ʒ ss.

Menthol, grs. iv.

Salammoniac, grs. 23 to 30.

M. Inhale.

Powders are best adapted to treating the nasal passages, as they remain longest in contact with the mucous membrane.

*For epistaxis*, the best new surgical remedy is the hot douche of 122° to 140° F. —*Deutsche med. Wochenschrift*, Jan. 24, 1889.

---

***Citrate of Caffeine in Eclampsia.***—B. Corney reports the case of a woman aged 23, who gave birth to an undersized but full-time child at 7 A.M. August 21st, after an easy labor of three hours. About noon headache came on; at 9 P.M. vomiting took place, and at 11 P.M. convulsions set in, lasting, with intermissions, ten hours. The bromides, hyoscyamus, chloral hydrate, and chloroform did not seem to have much effect. The patient remained in a deep stupor for three nights, and the two intervening days. There was slight fever, a weak pulse varying from 80 to 132, great cyanosis, incontinence of urine, and left hemoplegia. As the vital powers appeared to be rapidly failing, it was necessary to take some decisive step. Corney objected to alcohol, because of the imperfect aëration of the blood that was going on, as evinced by the cyanosis. He was uncertain how much of the stupor and cardiac weakness was due to the disease, and how much to the bromide and chloral. Caffeine suggested itself, and he immediately injected grs. iii. of the citrate dissolved with grs. iiss. of sodium salicylate in ♏x. of distilled water. This was followed in an hour by six grains more given by mouth, and two grains every two hours afterwards for six doses. Some general improvement took place, the pulse bettered, and the attacks of lividity ceased. The paralytic symptoms diminished in degree, and towards dawn on the 24th signs of returning consciousness were observed. The caffeine was continued for two days, and from this time the patient made a steady recovery. A week later the only remaining abnormalities were a certain degree of muscular weakness and debility. —*Practitioner*, Feb., 1889.

---

***Treatment of Vaginismus.***—Lutaud recommends, before resorting to operative treatment, to attempt the cure of this condition as follows: Introduce every night within the vulva a bougie composed of iodoform, gr. 15; extract of belladonna, gr. 7½; cacao butter, dr. 2½. Inject into the vagina three times daily a quart of hot water, to which has been added a teaspoonful of carbonate of soda, afterwards swabbing the vagina and vulva with a 10 per cent. solution of hydrochlorate of cocaine. This treatment is to be continued for a month, and attempts at coitus are to be practiced every two or three days after having anointed the vulva and penis with some lubricant. As parturition generally causes vaginismus to disappear, Lutaud advises that a hypodermic injection of $\frac{1}{6}$ of a grain of morphia should be given before coitus, as this, by its sedative action, may allow the act to be successfully performed, with pregnancy and the cure of the vaginismus as the result.—*Jour. de Méd. de Paris*, Nov. 18, 1888.

---

***Thymol in the Treatment of Tuberculosis.***—Dr. W. Philipowicz has administered thymol to 38 patients, 17 of whom were treated exclusively with this remedy, while the remaining 21 were given the preparation after diarrhœa had set in. The results of the first group were: 10 improvements and 4 deaths, while 3 remained under treatment. The results of the second group are not given, as the treatment was not a uniform one. Thymol was administered in daily doses of 45 grains, being given in gelatine capsules, in which medium the burning taste of the drug was less noticeable. Under the administration of thymol a diarrhœa will generally disappear in from two to four days, except when there exists amyloid degeneration of the intestinal walls. The drug can be taken for some time without producing any harmful effects: in fact, it seemed to the author that under its administration both the appetite and digestion were improved. In hæmoptysis it diminished the cough and the expectoration, it lowered the temperature, produced a gain in the body weight; in short a general improvement of the bodily condition, except in cases in which the disease was too far advanced.—*Wiener med. Presse*, Jan. 6, 1889.

---

***Extirpation of the Thymus Gland.***—In the *Finska Läkaresällskapets Handlingar* for November, 1888, are reported cases of partial extirpation of the gland for benign goître, made by Professor Af. Schulten, at Helsingfors. The operation as practiced in a series of cases extending from 1884-88 by different surgeons, had been invariably successful. Half the gland was removed in the three cases shown by Professor Af. Schulten, and he recommended removal of the degenerated portion only. The author remarks that in Finland goître is for the most part sporadic. The cases so grave as to threaten life by suffocation by compressing the trachea are not common. But when there are dyspnœa and more or less difficulty in swallowing the necessity of operation is indicated. In these cases treatment with iodine was of no avail. The author recommends in the operation the passing of the drainage-tube behind the sterno-mastoid. In reviewing the various treatments he shows the much smaller proportion, 3 per cent., of deaths in partial excision than in total excision, in which the mortality was 15 per cent. In rapidly growing goître in young persons he recommends ligature of all the thyroid arteries as practiced by Wölfler.—*The London Medical Recorder*, Jan. 21, 1889.

---

***The Contagiousness of Pneumonia.***—In a long article on this subject Netter[1] reviews the epidemics of pneumonia which have been recorded, and adds a few other instances which have come within his own experience. His most important conclusions are as follows:

1. Pneumonia is a contagious disease of parasitic origin, and is transmissible either directly or by the intervention of a third person, or by inanimate objects, such as wearing apparel, etc.

2. The pneumococci are not destroyed by desiccation, and are diffusible through the air, but not to great distances, at most the interval between three hospital beds. They maintain their virulence for a period which has not yet been definitely determined, but probably never more than three years.

3. Contagion is possible during the entire course of the disease and even after recovery.

4. The period of incubation averages from five to seven days, but may vary between one and twenty.

5. Patients who have passed through a pneumonia are dangerous both to themselves and their neighbors as living micrococci may be found in their saliva many years after. Thence in part the epidemic appearance of the disease in certain families during long periods, and also its frequent recurrence in certain individuals who have once survived it.

6. Rigid quarantine of the patients seems unnecessary, but other patients and healthy persons should not be brought into too intimate relations with them. The sick-room must be kept well ventilated and clean, the sputum disinfected, and the cocci lurking in the mouth destroyed so far as possible.—*Boston Medical and Surgical Journal.*

[1] Arch. Gén. de Méd. 1888, May-July.

---

***Magisterium Bismuthi in Infantile Summer Diarrhœa.***—In the St. Petersburg weekly *Rüsskaia Meditzina*, No. 30, 1888, Dr. A. Puginoff says that subnitrate of bismuth constitutes the most reliable remedy for epidemic summer diarrhœa in nurslings. He gives the drug in large doses, feeling sure that a pure preparation is excreted *per anum* wholly and in an unaltered state. Thus, to an infant of 4½ months, he administers 1½ or 2 grains every 2 hours. The main advantages of the subnitrate over all other means are stated to be these: 1. The drug does not give rise to any untoward accessory symptoms. 2. It is readily taken and perfectly well borne. 3. It acts on the intestinal tract both as a sedative and as an antiseptic.

---

***Tuberculosis from Contagion.***—At a meeting of the Finnish Medical Society at Helsingfors, Sept. 22, Mr. Runeberg reported a case of tuberculosis undoubtedly caused by contagion. The patient was a peasant, 39 years of age, who had an untainted family history, and showed in his own constitution no tendency to phthisis. Two years ago he was in perfect health; but the symptoms appeared a little after the death of his wife from consumption. He had occupied the same bed and nursed her during an illness of several years.—*The London Medical Recorder.*

---

***Gluten as an Article of Diet.***—Dr. Woltering strongly recommends (in the *Allgem. Med. Central-Zeitung*) the use of gluten as an article of diet, on account of its great nutritive qualities and its very low price. By means of tables of analyses he shows that pure gluten bread is about three times as nourishing as meat, and that bread made with 40 per cent. of pure gluten contains more albumen than the best hare or chicken.

